# Subclinical cardiac abnormalities and physical function in asymptomatic elderly

**DOI:** 10.1186/1532-429X-18-S1-P146

**Published:** 2016-01-27

**Authors:** Angela S Koh, Feiqiong Huang, Thu Thao Le, Jia ing Wong, Ru San Tan, Serene Chua, Yi Ying Han, Stuart Cook, Woon Puay Koh

**Affiliations:** 1 grid.419385.20000000406209905Cardiology, National Heart Centre Singapore, Singapore, Singapore; 2 grid.428397.30000000403850924Duke-NUS Graduate Medical School, Singapore, Singapore

## Background

While it is known that the heart remodels progressively with age, data characterizing the relationship between cardiac remodelling and function on physical function among the aged are limited.

## Aim

We investigated the association between left ventricular (LV) concentricity and LV function with handgrip strength (shown to correlate with mortality in the elderly) and timed-up-and-go test (for lower extremity function) among clinically asymptomatic elderly.

## Methods

In this community-based cohort of 28 patients free of known cardiac disease (mean age 73.8 ± 4) with preserved LV ejection fraction (mean LVEF 69.8 ± 6.4) and cardiac index (mean 3.1 ± 0.57), we assessed cardiac remodelling by cardiac MRI (concentricity^0.67^ (mass/end-diastolic volume^0.67^) and LV function by resting tissue Doppler imaging (TDI) performed at the septal and lateral mitral annulus, deriving myocardial systolic velocity (S), diastolic velocity (E) and ratio of E/A.

## Results

There were significant correlations between handgrip and LV concentricity (r = 0.49, p = 0.008), lateral S (r = 0.45, p = 0.041), septal A (r = 0.45, p = 0.04), and between timed-up-and-go and LV concentricity (r = 0.47, p = 0.013), lateral E/A (r=-0.49, p = 0.023), septal A (r = 0.45, p = 0.043). By regression analysis, LV concentricity (β=0.071, 95%CI 0.011-0.132, p = 0.023) (with adjustment for systolic blood pressure) and lateral E/A (β=-0.15, 95%CI -0.29—0.009, p = 0.039) were independently predictive of handgrip strength and timed-up-and-go respectively.

## Conclusions

These preliminary observations provide important insights into a possible link between subclinical alterations in cardiac structure and function and physical function, further study is required to clarify these findings with a view to preserve health and function amongst the elderly.

